# A nitric oxide-sensing two-component system regulates a range of infection-related phenotypes in *Burkholderia pseudomallei*

**DOI:** 10.1128/msphere.00423-25

**Published:** 2025-09-17

**Authors:** Matthew W. Scurlock, Stephen L. Michell, Steven L. Porter

**Affiliations:** 1 Biosciences, Faculty of Health and Life Sciences, University of Exeter 3286, , Exeter, United Kingdom; University of Wyoming, Laramie, Wyoming, USA

**Keywords:** melioidosis, nitric oxide, two-component regulatory systems, biofilms, *Burkholderia*, *pseudomallei*

## Abstract

The tier 1 bioterrorism agent *Burkholderia pseudomallei* causes melioidosis, a tropical disease with fatality rates that can exceed 40% despite antibiotic therapy. Antibiotic failure is likely to be, at least in part, due to biofilm-dwelling *B. pseudomallei,* and therefore, an improved understanding of how this pathogen regulates biofilm formation could reveal new opportunities for clinical intervention. The antimicrobial radical nitric oxide (NO) plays a key role in host immune defenses against bacteria, and the ability of *B. pseudomallei* to sense and mitigate NO toxicity is vital for establishing infection. NO-sensing proteins (NosPs), which have recently emerged as key regulators of biofilm formation in many bacterial species, use a FIST domain to sense NO via a bound heme. We hypothesized that the NosP homolog in *B. pseudomallei* would regulate biofilm formation and mediate NO-protective responses. We used [γ-^32^P]ATP autophosphorylation assays to show that NosP of *B. pseudomallei* controls the autophosphorylation rate of an associated histidine kinase protein (NosK) in an NO-dependent manner. NosK was found to phosphorylate a response regulator protein (NosR) with an HD-GYP output domain, which is associated with c-di-GMP signaling, therefore implicating NosP in modulating c-di-GMP-regulated phenotypes. Unmarked, in-frame deletion of either *nosP* or *nosK* caused significant changes in *B. pseudomallei* biofilm formation and increased sensitivity to nitrosative stress, in addition to affecting other virulence traits such as growth and swimming motility. These results indicate that NosP and NosK signaling control a range of infection-relevant phenotypes and may serve as targets for novel therapeutic intervention.

**IMPORTANCE:**

Melioidosis is an emerging, potentially life-threatening infection caused by the bacterium *Burkholderia pseudomallei*, killing ~89,000 people per year globally. Antibiotic therapy fails in ~10%–40% of cases, and hence, an improved understanding of the molecular mechanisms that control *B. pseudomallei* virulence could reveal new approaches for improving melioidosis treatment. Biofilm formation and resistance to the antimicrobial radical NO are virulence traits that help bacteria establish infections. Here, we show that two proteins in *B. pseudomallei*, NosP and NosK, work together to detect NO and regulate a suite of virulence traits, including NO resistance, biofilm formation, growth, and swimming motility. This work, therefore, improves our understanding of the molecular mechanisms that control infection-related phenotypes in *B. pseudomallei*.

## INTRODUCTION

*Burkholderia pseudomallei* is a Gram-negative, soil-dwelling saprophyte endemic to tropical and subtropical regions (1, 2). It is listed as a tier 1 bioterrorism select agent, being the etiological cause of melioidosis, an emerging and often fatal disease for which there is currently no vaccine (2). Antibiotic therapy frequently fails, with recurrence occuring in 5–28% of “treated” cases (2). Antibiotic failure is likely to be at least in part due to *B. pseudomallei* that dwell in biofilms: aggregates of surface-attached bacteria that exist within a self-secreted matrix of exopolysaccharides, lipids, proteins, and DNA (3, 4). Bacteria in biofilms are afforded protection from both antibiotics and host immune responses, therefore enabling pathogens to cause recurrent infections (5–7). Indeed, the minimum concentration of ceftazidime (a first-line antibiotic for melioidosis treatment) required to eliminate biofilm-dwelling *B. pseudomallei* is up to 1,000-fold more than the minimum inhibitory concentration of ceftazidime against planktonic *B. pseudomallei* (6). Hence, an improved understanding of how *B. pseudomallei* regulates biofilm formation could aid the development of anti-biofilm agents that sensitize *B. pseudomallei* to existing antibiotics.

Biofilm formation is promoted by high intracellular concentrations of the secondary messenger molecule cyclic di-GMP (c-di-GMP) (8). Intracellular levels of which are regulated by the opposing actions of diguanylate cyclases (DGCs), which utilize a GGDEF domain to synthesize c-di-GMP, and phosphodiesterases (PDEs), which use either an EAL domain or an HD-GYP domain to hydrolyze c-di-GMP (8). Metabolism of c-di-GMP in bacteria is often controlled by two-component systems (TCSs) (9). TCSs are typically comprised of two proteins: a stimulus-sensing histidine kinase (HK) and a partner response regulator (RR), which elicits downstream adaptational responses (10). HKs autophosphorylate on a conserved histidine residue, with the rate of autophosphorylation determined by sensing a signal (e.g., ligand binding). When autophosphorylated, the HK serves as a phosphodonor for its partner RR; the partner RR binds the autophosphorylated HK and catalyzes the transfer of the phosphoryl group from the conserved histidine residue of the HK onto a conserved aspartate residue within the RR (10). This activates the RR output domain to produce a physiological response, ultimately enabling bacteria to adapt to their environment (11). Hence, for RRs with c-di-GMP-metabolizing output domains, HKs can indirectly control c-di-GMP levels and therefore biofilm formation.

Most HKs are bifunctional, in that they can also adopt a phosphatase conformer, which hydrolyzes the phosphoryl group off their phosphorylated partner RR (12, 13). Signal sensing can either stimulate HKs to autophosphorylate or adopt a phosphatase-competent conformer (10, 13–15). The HK phosphatase conformer functions to rapidly terminate any phosphorylated RR signaling as soon as HK autophosphorylation has stopped, as well as suppressing any RR activation that may arise from non-specific phosphorylation by other HKs (10, 13). The ratio of autophosphorylation to phosphatase activity of a given HK therefore dictates the level of phosphorylation of its partner RR and ultimately the extent of the response elicited by a TCS at any one time.

HK autophosphorylation activity can be controlled by interactions with auxiliary sensor proteins. One type of auxiliary sensor protein is the nitric oxide-sensing protein (NosP). NosP homologs contain a FIST domain, which binds a heme moiety that detects nitric oxide (NO). These proteins are often encoded in operons with a HK protein (NosK) and a RR protein with a c-di-GMP-metabolizing output domain (NosR). Following autophosphorylation, NosK-P serves as a phosphodonor for NosR, which modulates the activity of NosR’s c-di-GMP-metabolizing output domain. NosPs control the autophosphorylation rate of NosK proteins in an NO-dependent manner and thereby indirectly regulate c-di-GMP-dependent phenotypes in response to NO (16–19). Indeed, NosPs have been shown to control biofilm formation in many species such as *Burkholderia thailandensis*, *Pseudomonas aeruginosa, Shewanella oneidensis,* and *Legionella pneumophila* (16–20).

NO itself is an extremely reactive radical and is synthesized to antimicrobial, micromolar concentrations by host inducible NO synthase (iNOS) enzymes as part of the innate immune response (21, 22). NO can exert toxicity directly toward pathogens by reacting with their DNA, proteins, and lipids, or indirectly by reacting with molecular oxygen or superoxide to form more potent antimicrobial reactive nitrogen species such as the bactericidal peroxynitrite (ONOO^-^) (23, 24). Although many of the mechanisms that *B. pseudomallei* employs to mitigate NO toxicity during infection are well characterized (25–28), the mechanisms by which *B. pseudomallei* detects NO are poorly understood.

We hypothesized that a NosP homolog could regulate biofilm formation and mediate NO-protective responses in *B. pseudomallei*. We therefore searched for NosP homologs in *B. pseudomallei* K96243 and identified BPSS1647 (NosP), which contains a FIST domain. NosP was shown to modulate the autophosphorylation rate of the adjacently encoded HK BPSS1646 (NosK) in an NO-dependent manner. Unmarked, in-frame deletion mutants of the *nosP* or *nosK* genes displayed drastic changes in infection-related phenotypes, including biofilm formation, swimming motility, and resistance to nitrosative stress, therefore implicating this TCS in the regulation of *B. pseudomallei* virulence.

## RESULTS

### Identification of a NosP-encoding operon in *B. pseudomallei*

**Fig 1:**
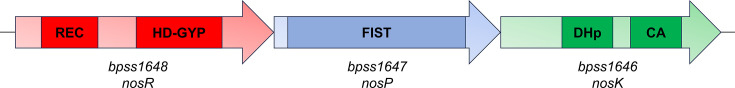
Structure of the *nosRPK* operon and the predicted domain composition of each protein. NosR contains a phosphoryl-receiving REC domain and a degenerate c-di-GMP-hydrolyzing HD-GYP output domain, where its HD-GYP motif is altered to HG-GTP, which likely makes it catalytically inactive. NosP has a heme-binding FIST domain, which can sense NO. NosK has both domains needed for autophosphorylation (DHp and CA domains) but has no recognized sensory domain.

To identify a NosP homolog in *B. pseudomallei*, we searched for proteins predicted to contain a FIST domain in the clinical isolate strain *B. pseudomallei* K96243 using EMBL-Smart and by performing BLASTP searches with PA1975 (a NosP from *P. aeruginosa*). These returned BPSS1647 (NosP) as the only hit (Fig. 1), a single domain protein encoded in an operon with a HK, BPSS1646 (NosK), and a RR, BPSS1648 (NosR). In common with other HKs, NosK harbors a dimerization and histidine phosphotransfer (DHp) domain, and a catalytic and ATP-binding (CA) domain (Fig. 1). However, there are no predicted transmembrane regions in NosK, and it contains no recognized sensory domain, despite having ~130 amino acids N-terminal to the DHp domain in a position where a sensory domain is commonly found in other HKs. Because NosK lacks an intrinsic sensory domain, we hypothesized that NosP may act as an auxiliary sensor protein for NosK by modulating its autophosphorylation rate in an NO-dependent manner. NosR has an N-terminal receiver (Rec) domain, which contains the conserved phosphoryl-accepting aspartate residue, and a C-terminal HD-GYP domain (Fig. 1) (29). Proteins with HD-GYP domains are c-di-GMP PDEs, but the catalytic HD-GYP motif of NosR is altered to HG-GTP (8). The HD residues are critical for coordinating metal ions necessary for c-di-GMP hydrolysis, and therefore, it is likely that NosR is a degenerate HD-GYP domain protein (30). Nonetheless, degenerate HD-GYP proteins have been found to influence c-di-GMP metabolism by interacting with and regulating the activity of other bona fide c-di-GMP-metabolizing proteins (31).

### NosP inhibits NosK autophosphorylation

**Fig 2:**
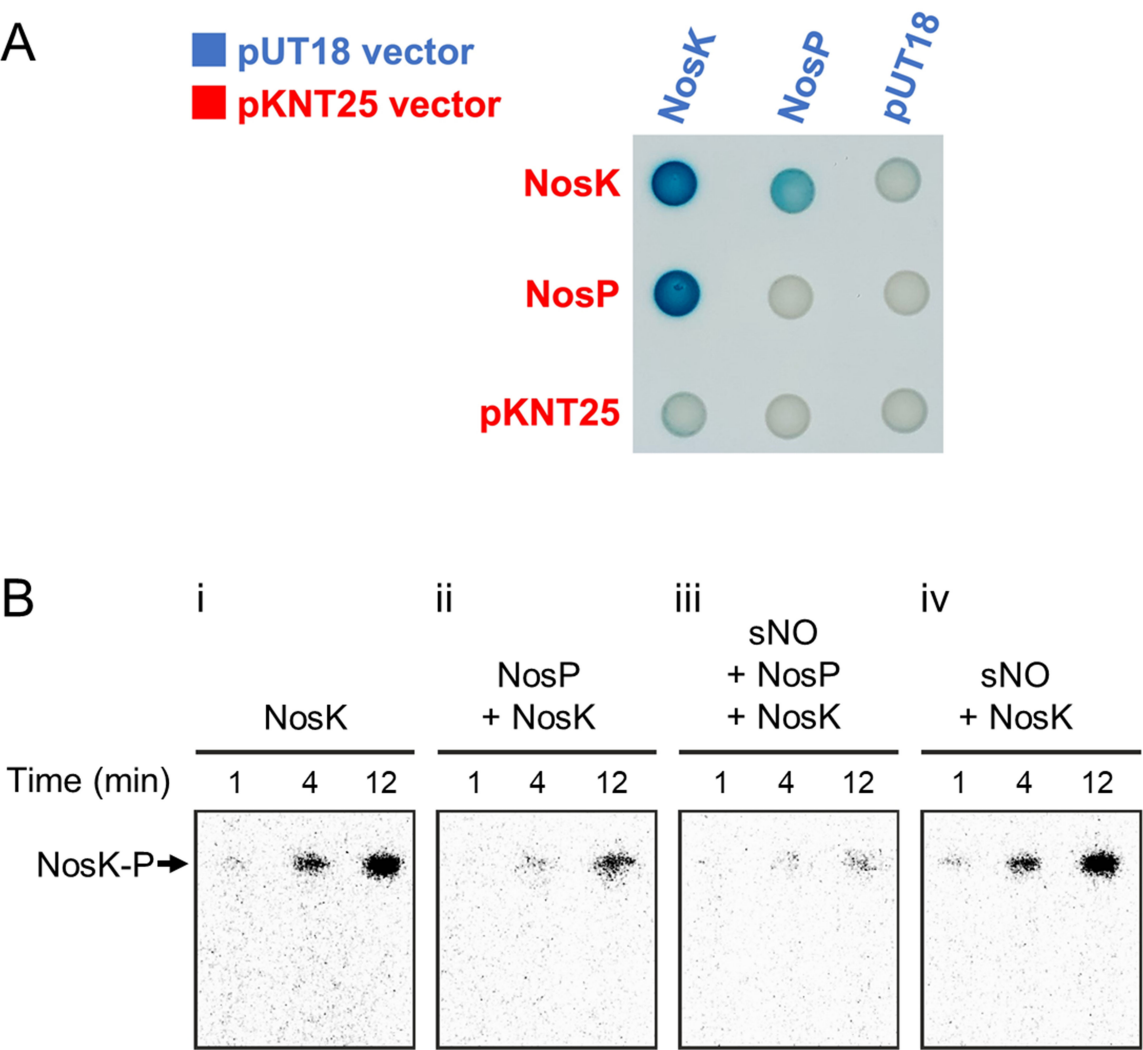
NosP inhibition of NosK autophosphorylation is potentiated by NO. (**A**) Two-hybrid assay showing that NosK homodimerizes and interacts with NosP. (**B**) NosK [γ-^32^P]ATP autophosphorylation assays. (i) NosK alone, (ii) NosK incubated with NosP, (iii) NosK incubated with spermine NONOate (sNO) and NosP, and (iv) NosK incubated with spermine NONOate only. [NosK] = 12.5 µM, [NosP] = 75 µM, [[γ-^32^P]ATP] = 0.5 mM, [spermine NONOate] = 1 mM.

Using a bacterial adenylate cyclase two-hybrid assay (32), we found that NosP binds to NosK (Fig. 2A). Autophosphorylation assays using [γ-^32^P]ATP and purified protein showed that hemin-bound NosP inhibits NosK autophosphorylation (Fig. 2Bi and Bii). Comparable results have been seen in *Burkholderia thailandensis,* where the hemin-bound homolog of *B. thailandensis* NosP, *Bth_ii0732*, inhibited the autophosphorylation of the homolog of *B. thailandensis* NosK, *Bth_ii0733* (19).

### The NO-donor molecule spermine NONOate enhances NosP-mediated inhibition of NosK autophosphorylation

To investigate how NO may modulate NosP-mediated regulation of NosK autophosphorylation, the NO-donor molecule spermine NONOate was added to NosK and NosP. One mole of spermine NONOate dissociates to release 2 moles of NO, with a half-life of ~39 minutes at 37°C. The addition of spermine NONOate potentiated NosP-mediated inhibition of NosK autophosphorylation as observed by a decreased NosK-P band intensity when spermine NONOate, NosP, and NosK were coincubated (Fig. 2Biii). To confirm that this increased inhibition of NosK autophosphorylation was NosP-dependent and not due to NO-induced denaturation of NosK, spermine NONOate was also incubated with only NosK without NosP (Fig. 2Biv), which showed that autophosphorylation of NosK was unaffected by the presence of spermine NONOate alone. These results show that spermine NONOate enhances the ability of NosP to inhibit NosK autophosphorylation, which is consistent with NosP acting as the auxiliary sensor protein for NosK.

### NosK phosphorylates NosR

**Fig 3:**
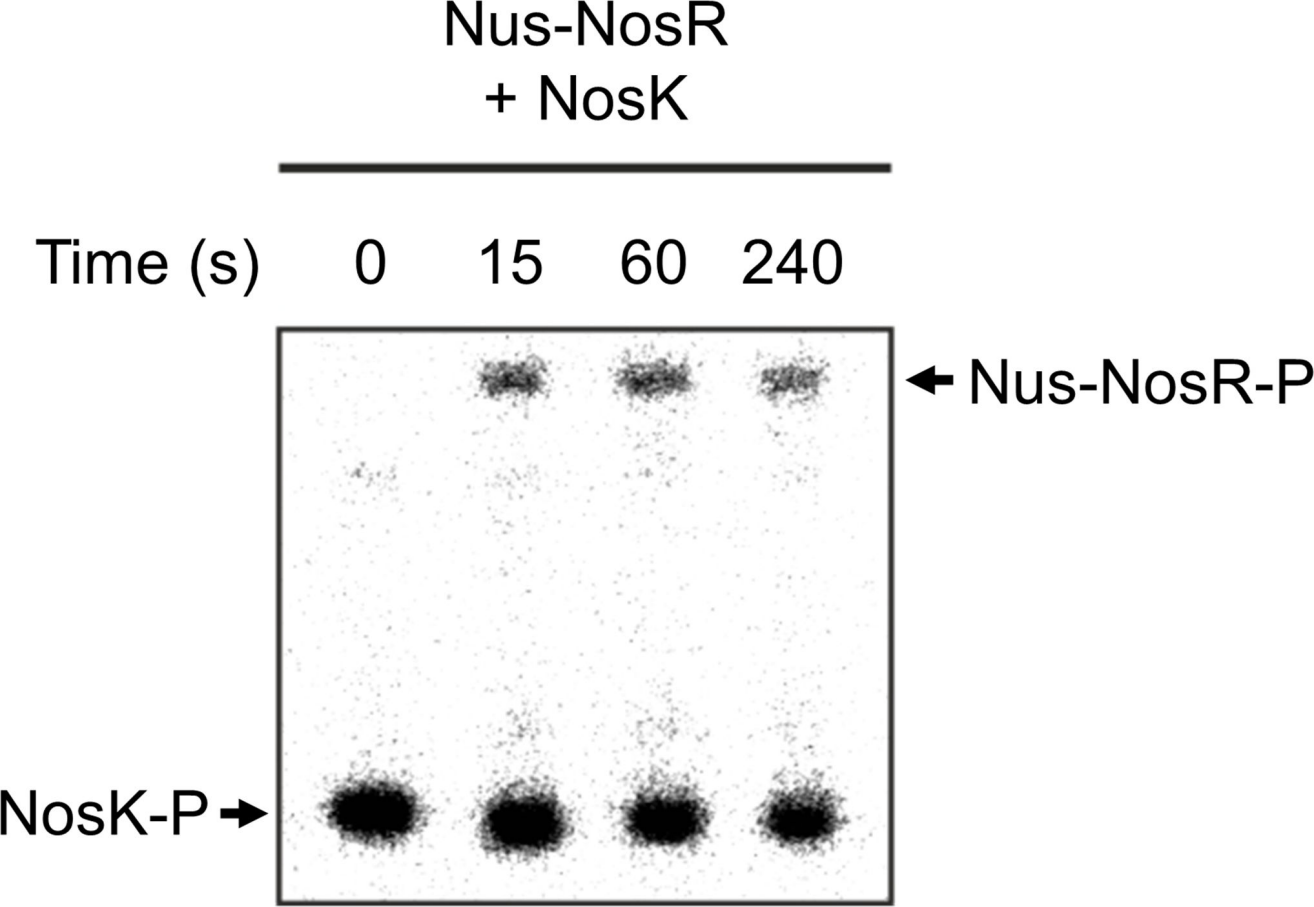
NosK phosphorylates NosR. [NosK] = 65 µM, [Nus-NosR] = 5 µM, [[γ-^32^P]ATP] = 2 mM. NosK was preincubated with ATP for 45 min prior to the addition of Nus-NosR at time = 0.

The partner RR for a HK is often encoded in the same operon. We therefore hypothesized that NosR (BPSS1648) would be the partner RR for NosK and tested this using phosphorylation assays. Due to the similar molecular weights of NosK (44.2 kDa) and NosR (53.3 kDa), a NusA tag was added to NosR (Nus-NosR = 114.4 kDa) to help differentiate between any potential ^32^P-labeled protein bands. NosK was found to rapidly phosphorylate NosR, as shown by the appearance of a Nus-NosR-P band at 15 seconds (Fig. 3), indicating that they work together as a TCS. The rapid rate of this phosphotransfer reaction, coupled with both proteins being encoded together by the same operon, implies that NosR is the physiological phosphotransfer partner of NosK.

### NosK and NosP signaling affects growth in nutrient-limited environments

We then investigated how this NO-responsive TCS regulates *B. pseudomallei* K96243 behaviour. Although two independent transposon mutagenesis screens have identified either *nosP* (*bpss1647*) (33) or *nosK* (*bpss1646*) (34) as genes required for *B. pseudomallei* growth, transposon insertion methods can be liable to false positives, and hence, we initially pursued making unmarked in-frame deletion mutants. We succeeded in making unmarked deletions of both genes (Fig. S1A and B), showing that neither gene is essential for *B. pseudomallei* K96243 growth.

**Fig 4:**
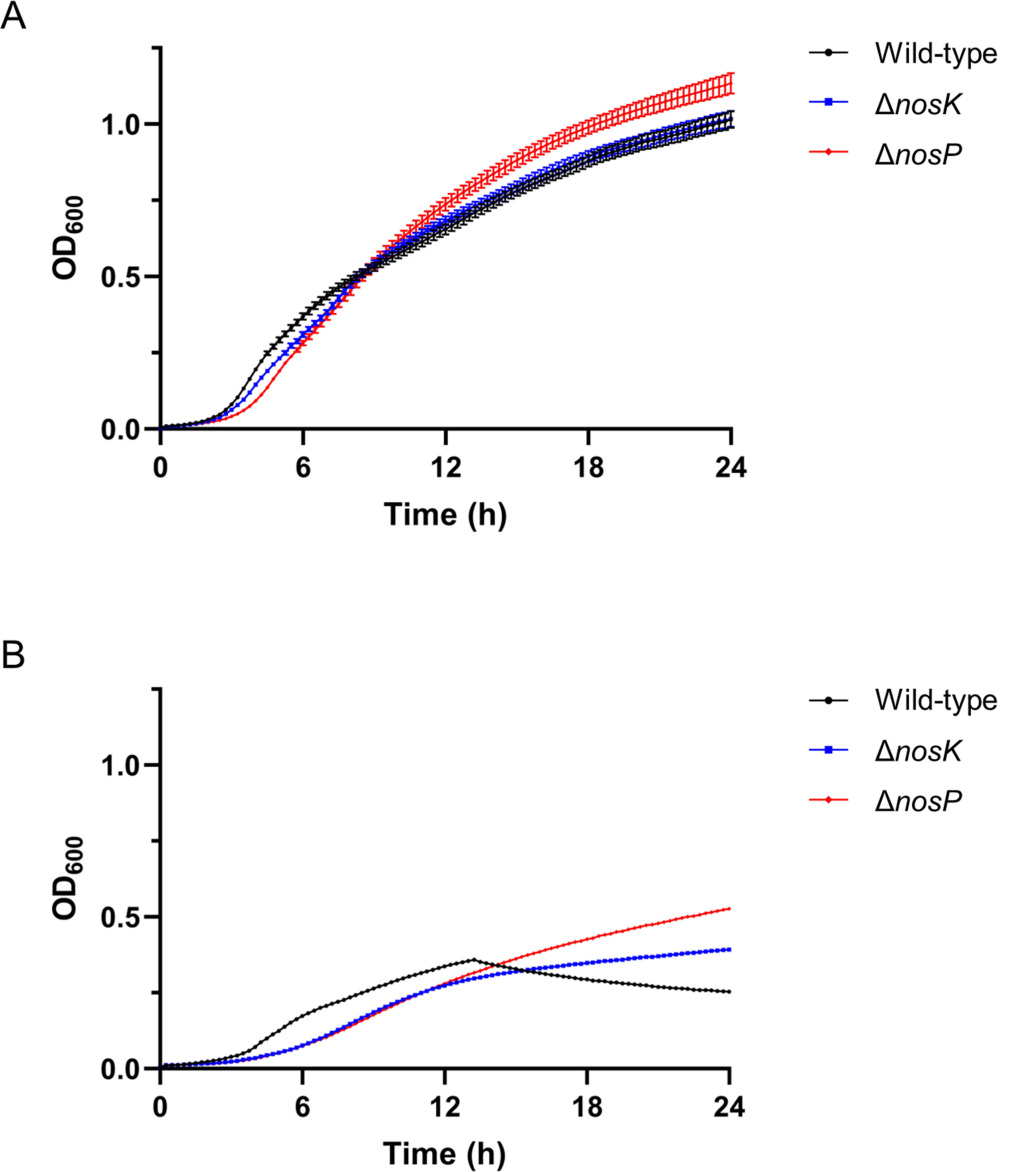
NosK and NosP regulate *B. pseudomallei* growth in nutrient-limited environments. Growth of *B. pseudomallei* K96243 wild type, Δ*nosK,* or Δ*nosP* in (**A**) LB media or (**B**) M9SMM. Data are the mean of either 10 (**A**) or 7 (**B**) replicates at each time point, with error bars representing ± SEM.

We first assayed for potential changes in growth rate between the wild-type and mutant strains. Wild-type, Δ*nosK,* and Δ*nosP* strains were first grown in LB media, which showed that the growth rates of the two mutants were similar to the wild type, although the Δ*nosP* strain grew to a higher OD_600_ (Fig. 4A). The three strains were then grown in nutrient-limited M9 minimal media supplemented with 20 mM succinate (M9SMM). The wild-type strain had an unusual growth curve in M9SMM, with the OD_600_ initially rising but then falling at around 13 h (Fig. 4B). This fall was reproducibly seen in M9SMM but never seen in LB media. We do not know the cause of this fall, but it may be linked to nutrient stress. The wild-type strain exited lag phase ~3 h earlier than the mutants, and this would lead to faster depletion of the nutrients. Nutrient limitation itself may have caused the drop, or it may have caused one of the prophages present in the K96243 genome to switch from lysogenic to lytic mode (35, 36). Unlike the wild-type strain, the Δ*nosK* or Δ*nosP* mutant strains did not show this fall, suggesting that their initially slower growth may have had the later benefit of having conserved enough nutrients to avoid the OD_600_ drop seen in the wild-type strain. Growth of the Δ*nosK* mutant is slightly different from that of the Δ*nosP* mutant in minimal media, and this is statistically significant (Fig. 4B). *nosK* deletion eliminates NosK activity completely, whereas *nosP* deletion leaves NosK with its basal level of activity. As the level of NosK activity differs between the mutants (no activity vs basal activity), this could explain their slightly different growth rates. Overall, these data show that although the Δ*nosK* and Δ*nosP* strains have very similar growth profiles to the wild-type strain in rich (LB) media, they have very different profiles in nutrient-limited M9SMM, suggesting NosP and NosK signaling affect growth in nutrient-limited environments.

### NosK and NosP regulate biofilm formation

**Fig 5:**
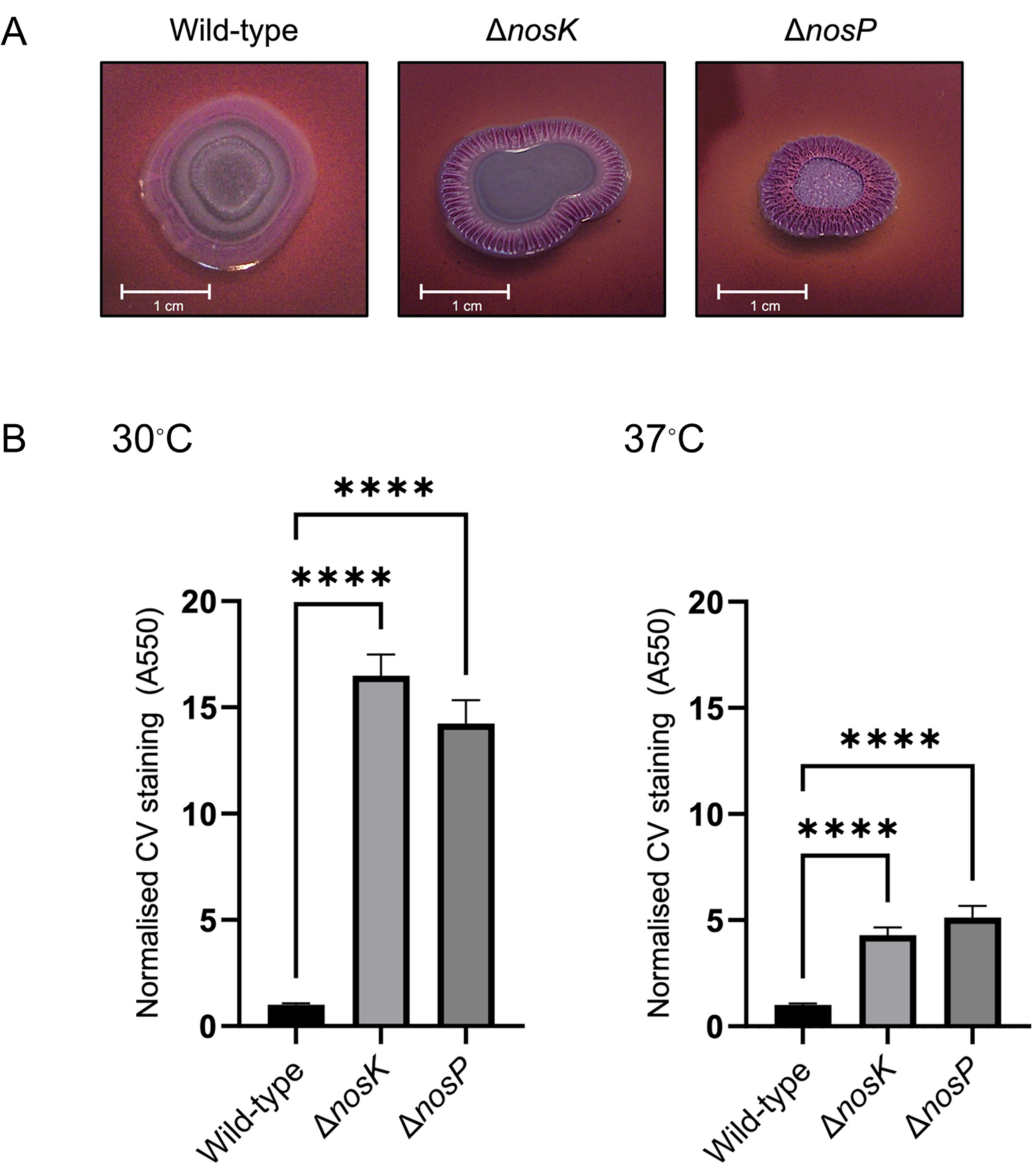
NosK and NosP regulate biofilm formation. (**A**) Representative photographs of *B. pseudomallei* wild-type, Δ*nosK,* and Δ*nosP* biofilm morphology on Congo red agar plates. (**B**) Both *B. pseudomallei* mutant strains form more biofilm on peg-lids than the wild type at 30°C and 37°C. The data are the mean, and error bars show the SEM from at least 25 technical replicates from at least three independent experiments. Data analysis was performed in GraphPad Prism using a Kruskal-Wallis test with multiple comparisons, ****P < 0.0001. CV, crystal violet. The raw data for these graphs are presented in Table S2.

An improved understanding of *B. pseudomallei* biofilm formation could reveal new opportunities for clinical intervention. NosP and NosK proteins have been found to regulate biofilm formation in several species (16–18). To qualitatively assess if NosP and NosK regulate biofilm formation in *B. pseudomallei*, we used a Congo red biofilm morphology assay. Both the Δ*nosK* and Δ*nosP* strains bound more Congo red dye than the wild-type strain (shown by a deeper color) and, unlike the wild-type strain, had wrinkly colony morphologies, both indicators of increased biofilm formation in the mutant strains (Fig. 5A).

To quantify changes in biofilm formation, a peg-lid 96-well plate biofilm assay was performed. Biofilm formation was tested at both 30°C and 37°C because previous studies found *B. pseudomallei* biofilm formation to be thermoregulated (37). The Δ*nosK* and Δ*nosP* strains formed ~16- and ~14-fold more biofilm than the wild-type strain, respectively, at 30°C, and ~4-fold and ~5-fold more biofilm than the wild-type strain, respectively, at 37°C (Fig. 5B). Taken together, the Congo red biofilm assay and the 96-well plate biofilm assay show that NosP and NosK regulate *B. pseudomallei* biofilm formation.

### NosK and NosP regulate swimming motility

**Fig 6:**
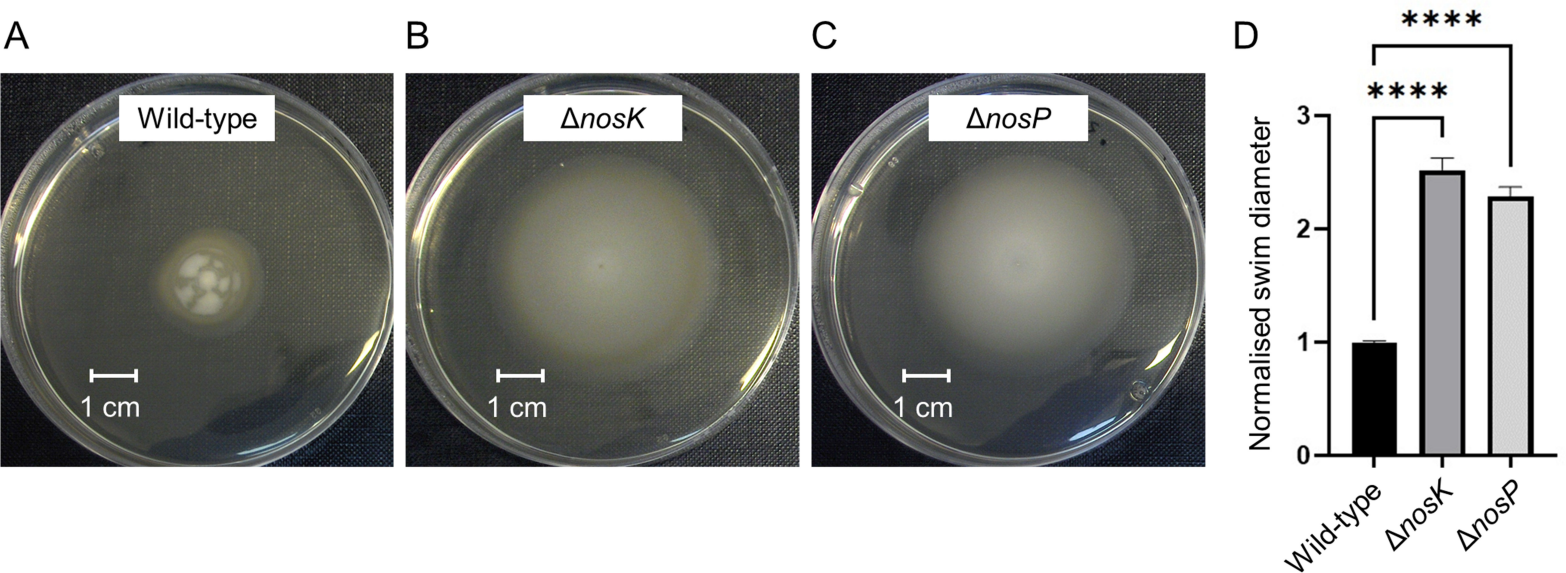
*B. pseudomallei* Δ*nosK* and Δ*nosP* swim significantly further than the wild-type strain. (**A–C**) Representative photograph of *B. pseudomallei* K96243 wild type, Δ*nosK,* and Δ*nosP* swimming motility on LB solidified with 0.3% agar after 48 h at 37°C. (**D**) Comparison of *B. pseudomallei* K96243 wild type, Δ*nosK,* and Δ*nosP* mutant swimming diameter after 48 h of incubation. Data are the mean of at least nine technical replicates from at least three independent experiments, with error bars representing the SEM. Significance values were obtained by performing a one-way ANOVA with multiple comparisons, ****P < 0.0001.

Swimming motility is another virulence trait regulated by c-di-GMP levels, enabling dissemination from an initial site of infection (38). We hypothesized that NosK and its regulator NosP may control swimming motility. Assays using semi-solid LB agar plates showed that the Δ*nosK* and Δ*nosP* strains swim significantly further than the wild-type strain (Fig. 6A through D). It was surprising to find that both swimming motility and biofilm formation were upregulated in the mutant strains, as these two phenotypes are widely considered to be inversely regulated by c-di-GMP levels (8).

### NosK and NosP regulate NO resistance

**Fig 7:**
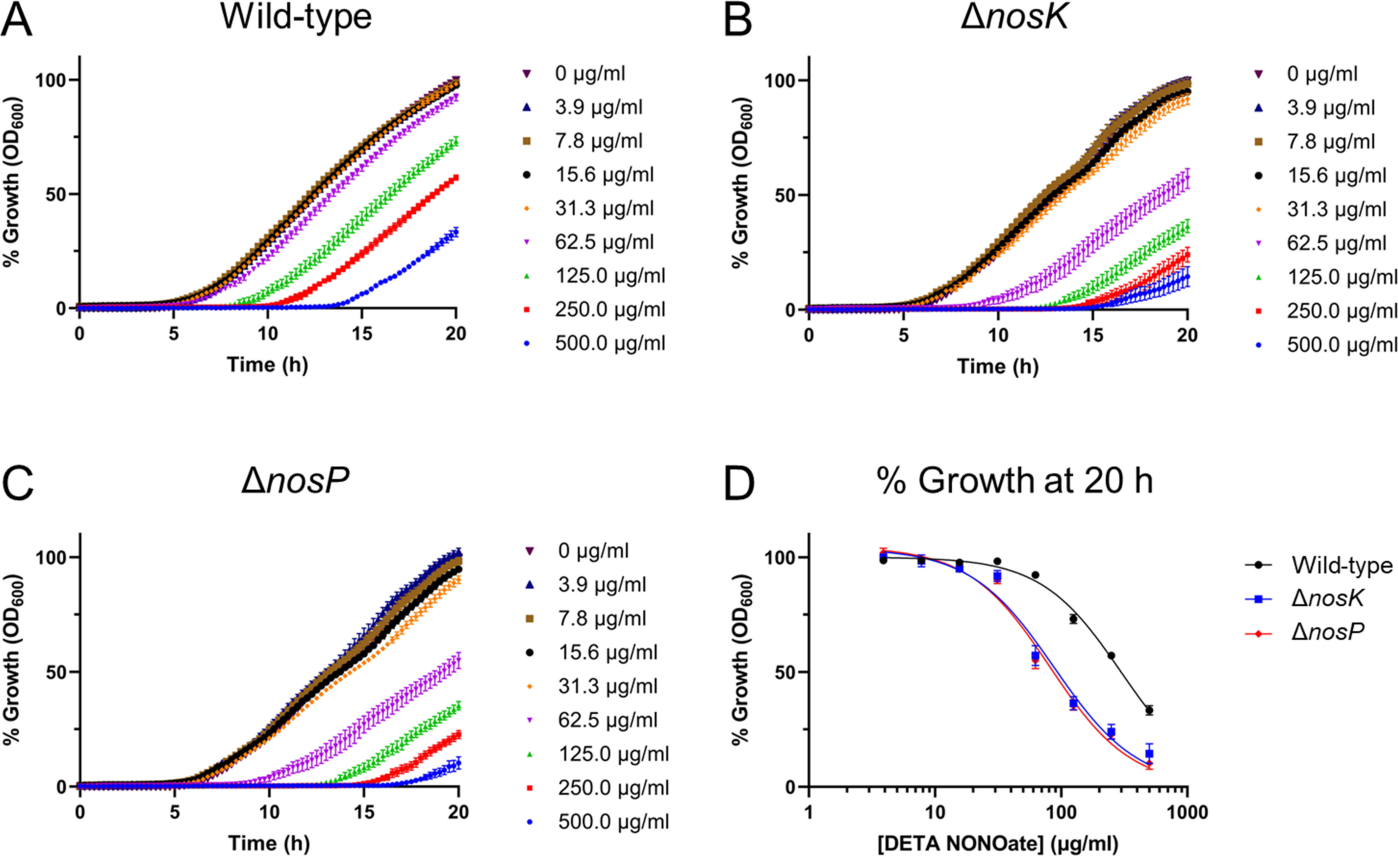
The *B. pseudomallei* Δ*nosK* and Δ*nosP* strains are more sensitive to DETA NONOate growth inhibition than the wild-type strain in MHB. (**A–C**) Percentage growth of the wild-type, Δ*nosK,* and Δ*nosP* strains in MHB supplemented with varying concentrations of DETA NONOate relative to the growth of the corresponding strain at 20 h without DETA NONOate. (**D**) DETA NONOate dose-response curve of the three strains in MHB after 20 h. Data are the mean of six technical replicates from three independent experiments, with error bars representing ± SEM.

As NosK and NosP constitute an NO-responsive signaling pathway, we considered the possibility that NosK and NosP may mediate NO-protective responses. To test this, *B. pseudomallei* wild-type, Δ*nosK,* and Δ*nosP* strains were challenged with the NO-donor molecule diethylenetriamine (DETA) NONOate. DETA NONOate decomposes to liberate 2 moles of NO per mole of parent compound with a half-life of 20 h at 37°C, pH 7.4, therefore providing a sustained release of NO into the growth media. *B. pseudomallei* strains were challenged with varying DETA NONOate concentrations in Mueller-Hinton broth (MHB) for 1 half-life (20 h in the conditions used [37°C, pH 7.4]). We first characterized the growth of the wild-type, Δ*nosK,* and Δ*nosP* strains in drug-free MHB and found that the three strains have similar growth profiles in this nutrient-rich medium (Fig. S2). The Δ*nosK* and Δ*nosP* mutant strains were then found to be ~3.4-fold and ~3.7-fold more sensitive to DETA NONOate, respectively, than the wild-type strain (Fig. 7A through D; Table 1). To confirm that the growth inhibition observed is due to the NO produced and not the DETA, all three strains were grown in MHB containing 3.1 mM DETA (500 µg/mL DETA NONOate is equivalent to 3.1 mM). As the growth of all three strains was unhindered by DETA, this confirms the DETA NONOate-mediated growth inhibition is NO-dependent (Fig. S3A through C).

**TABLE 1:** DETA NONOate IC_50_ values against *B. pseudomallei* K96243 wild-type, Δ*nosK,* and Δ*nosP* strains in different media^*a*^

Strain	Mueller Hinton broth			M9 succinate minimal media		
	IC_50_ (μg/mL)	Fold change	95% CI	IC_50_ (μg/mL)	Fold change	95% CI
Wild type	298	1	279–319	146	1	123–165
Δ*nosK*	88	3.4	78–105	19	7.7	17–40
Δ*nosP*	81	3.7	75–95	18	8.1	12–23

^*a*^ The fold change is relative to the wild-type IC_50_ value. CI, confidence interval.

### The increase in NO susceptibility of th*e B. pseudomallei* Δ*nosK* **and** Δ*nosP* strains is greater in M9SMM than MHB

**Fig 8:**
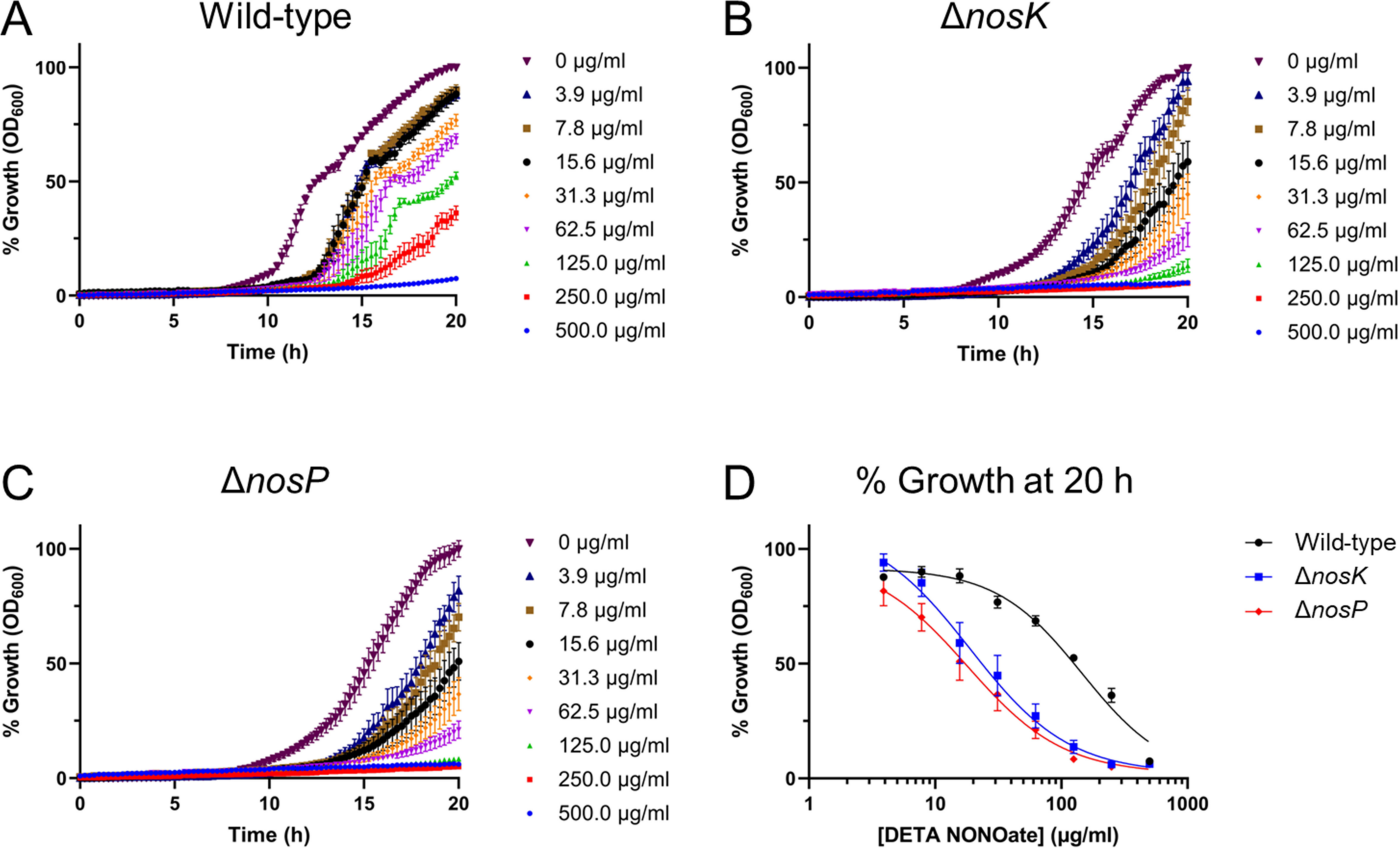
The *B. pseudomallei* Δ*nosK* and Δ*nosP* strains are more sensitive to DETA NONOate growth inhibition than the wild-type strain in M9SMM. (**A–C**) Percentage growth of the wild type, Δ*nosK,* and Δ*nosP* strains in M9SMM supplemented with varying concentrations of DETA NONOate relative to the growth of the corresponding strain at 20 h without DETA NONOate. (**D**) DETA NONOate dose-response curve of the three strains in M9SMM after 20 h. Data are the mean of eight technical replicates from four independent experiments, with error bars representing ±SEM.

Compared with the wild-type strain, the Δ*nosK* and Δ*nosP* strains grew to a higher OD_600_ in M9SMM (Fig. 4B). Having shown that in MHB, the Δ*nosK* and Δ*nosP* strains are ~3.4-fold and ~3.7-fold more sensitive to DETA NONOate than the wild type, respectively (Fig. 7A through D), we repeated the DETA NONOate challenge in M9SMM. Interestingly, after 20 h of incubation, the DETA NONOate IC_50_ value for the wild-type strain was 146 µg/mL, which was ~7.7-fold and ~8.1-fold greater than the DETA NONOate IC_50_ values for the Δ*nosK* and Δ*nosP* strains, respectively (Fig. 8A through D, Table 1). Hence, the difference in DETA NONOate susceptibility between the *B. pseudomallei* wild-type and Δ*nosK* or Δ*nosP* strains when challenged in M9SMM is even greater than when challenged in MHB. The growth of all three strains in M9SMM was not inhibited by the addition of 3.1 mM DETA, thereby confirming that the DETA NONOate-mediated growth inhibition is NO-dependent (Fig. S4A through D).

## DISCUSSION

NO resistance and biofilm formation are key virulence traits that help bacteria to withstand immune responses and colonize hosts. In this study, we showed that a NosP homolog in *B. pseudomallei*, and its partner HK, NosK, work together to detect NO and control phenotypes associated with virulence, such as resistance to nitrosative stress, biofilm formation, growth in nutrient-limited environments, and swimming motility.

### NosK and NosP mediate resistance to NO

Given their function in detecting NO, we investigated whether the loss of NosK or NosP would affect the sensitivity of *B. pseudomallei* to this antimicrobial radical. Indeed, the NO-donor molecule DETA NONOate inhibited the growth of the Δ*nosK* and Δ*nosP* strains more than the wild-type strain, showing that NosK and NosP mediate physiological responses that confer resistance to NO.

It was interesting to note the extent of the difference in DETA NONOate sensitivity between the wild-type and mutant strains depending on the growth media; the Δ*nosK* and Δ*nosP* strains were ~3.4-fold and ~3.7-fold more sensitive, respectively, to DETA NONOate than the wild type in nutrient-rich MHB (Fig. 7) but were ~7.7-fold and ~8.1-fold more sensitive than the wild type when grown in nutrient-limited M9SMM (Fig. 8). This increase in NO-susceptibility in M9SMM compared with MHB could be related to the unusual growth profile seen for the wild-type strain in M9SMM as neither mutant strain showed the abrupt drop in OD_600_ seen in the wild-type strain (Fig. 4B). Hence, NosK and NosP signaling may regulate a growth-limiting stress response induced by nutrient-limited conditions, and the loss of this function in the Δ*nosK* or Δ*nosP* mutants may contribute toward their increased DETA NONOate sensitivity in M9SMM.

### NosK likely functions primarily as a phosphatase *in vivo*

Deletion of either *nosK* or *nosP* caused similar changes in phenotype. If NosK functioned solely as a kinase for NosR, then *nosK* deletion would result in less NosR-P. NosP was shown to inhibit NosK autophosphorylation, thus the deletion of *nosP* would cause increased NosK autophosphorylation and therefore more NosR-P. Hence, deletion mutants of *nosK* and *nosP* would be expected to have opposing changes in NosR-P levels and therefore contrasting phenotypes. However, the two mutant strains exhibited similar changes in phenotype in a variety of assays, which is inconsistent with NosK functioning solely as a kinase.

**Fig 9:**
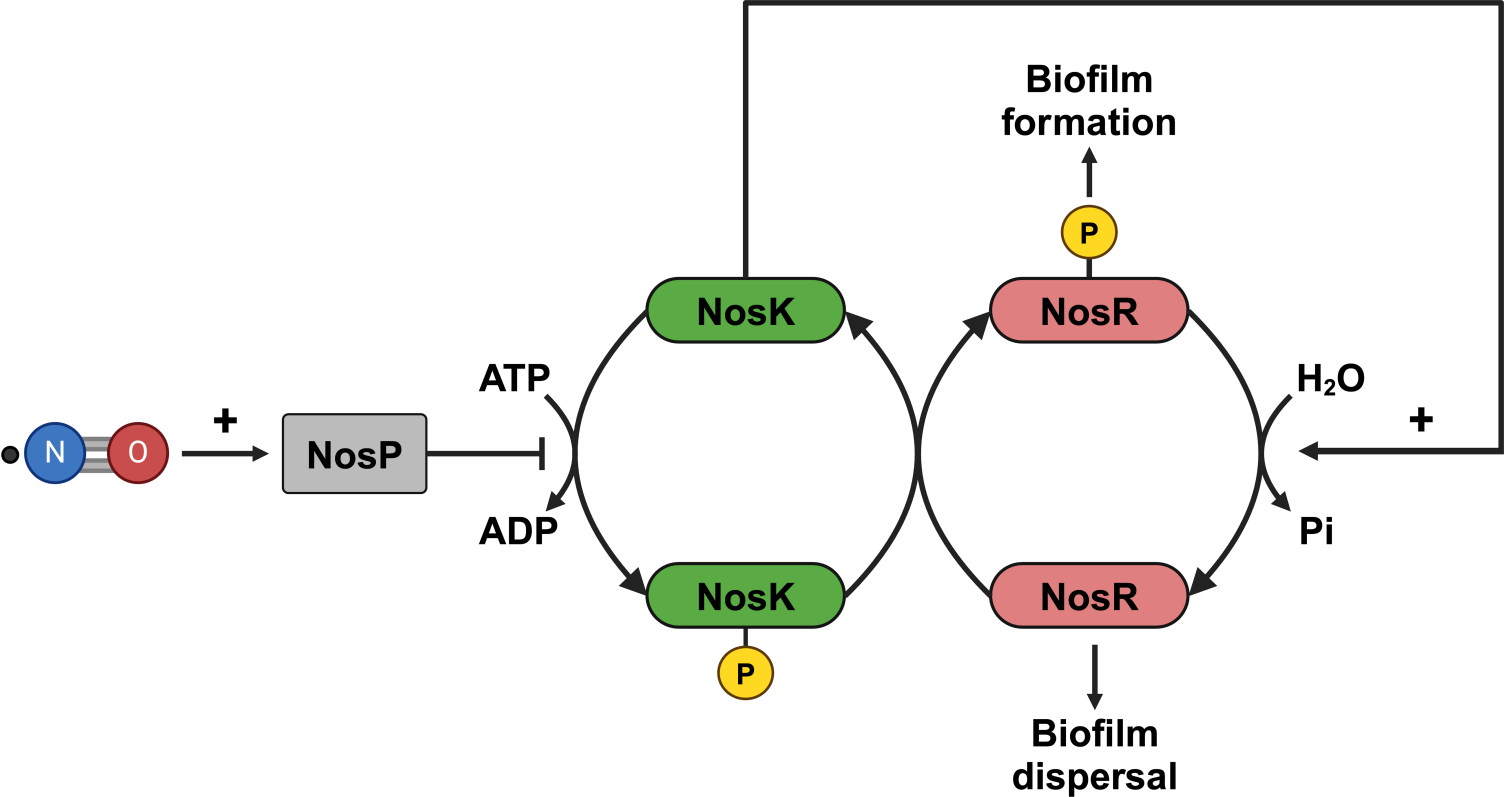
Model in which NosP inhibits NosK autophosphorylation and promotes NosK to dephosphorylate NosR-P. Sensing of NO by NosP potentiates NosP-mediated inhibition of NosK autophosphorylation. Deletion of *nosK* or *nosP* increases NosR-P levels, as it can be generated by either NosK (in Δ*nosP*) or other HKs/small-molecule-phosphodonors (in Δ*nosK*). Despite its degenerate HD-GYP domain, we hypothesize that NosR-P increases c-di-GMP levels, and therefore biofilm formation, either by inhibiting a bona fide PDE or by activating a DGC. Created in BioRender (S.Porter, 2025, https://BioRender.com/5f7wzr1).

Many HKs are bifunctional, having both kinase and phosphatase activity that are reciprocally regulated; HKs with phosphatase activity have a conserved phosphatase motif (H-E/D-x-x-T/N) in their DHp domain (39, 40). As NosK matches this consensus (with H-E-I-N-N), it is likely to be bifunctional and capable of dephosphorylating as well as phosphorylating its RR substrate, NosR (Fig. 9). Under conditions favoring NosK phosphatase activity, its deletion would result in increased levels of NosR-P. This is because non-specific, or possibly even specific, phosphorylation of NosR by other HKs or small molecule phosphodonors would result in the accumulation of NosR-P due to the loss of NosK phosphatase activity. Similarly, deletion of *nosP* would switch the balance of kinase/phosphatase activity of NosK in favor of kinase, increasing the production of NosR-P. Therefore, under conditions where NosK phosphatase activity dominates over kinase activity in the wild-type strain, the effects of deleting either *nosK* or *nosP* would be similar, both leading to elevated levels of NosR-P. Indeed, both strains have similar changes in growth profiles, biofilm formation, swimming motility, and NO-resistance compared with the wild-type strain. Thus, it can be inferred that under these assay conditions, NosK primarily acts as a phosphatase in the wild-type strain.

### Increased swimming and biofilm formation by the Δ*nosK* and Δ*nosP* mutants

The Δ*nosK* and Δ*nosP* mutants have considerably increased biofilm formation (Fig. 5) and swimming motility (Fig. 6). In many bacteria, these phenotypes are inversely regulated, with biofilm formation generally favored by high c-di-GMP concentrations, whereas low c-di-GMP concentrations are associated with swimming motility (8). However, this simple model is complicated by the role of flagella in biofilm formation; flagella have been found to not only facilitate movement toward a surface but also mediate surface adhesion and mechanosensing of surface contact in the initial stages of biofilm formation (38, 41, 42). Furthermore, flagella have also been implicated in contributing toward the biofilm mechanical structure (43). Additionally, a study of eight different *E. coli* strains found that those that formed the most biofilm were also the most motile and showed higher expression of genes involved in motility (44). This could explain how the *B. pseudomallei* Δ*nosK* or Δ*nosP* mutants have both increased motility and enhanced biofilm formation.

### Comparison with NosP signaling in other bacteria

NosP signaling has been investigated in other bacterial species. Similar to Fu et al.’s study on NosP in the closely related strain *B. thailandensis* E264 (19), we found that in *B. pseudomallei*, NosP interacts with NosK and inhibits its autophosphorylation and that NosK-P is a phosphodonor for NosR. However, our finding that NO potentiates NosP-mediated inhibition of NosK autophosphorylation in *B. pseudomallei* contrasts with Fu et al.’s finding that NO relieves NosP-mediated inhibition of NosK autophosphorylation in *B. thailandensis* (19). The effect of NO on NosP-mediated inhibition of NosK varies between species. In some species, NO potentiates inhibition (*B. pseudomallei*, *P. aeruginosa*, *V. cholera*), whereas in other species, NO relieves NosP inhibition (*B. thailandensis*, *L. pneumophila*, *S. oneidensis*) (16–19, 45). The differing effect of NO on NosP-mediated inhibition of NosK between *B. thailandensis* and *B. pseudomallei* could reflect their difference in sensitivity to this radical; *B. thailandensis* is more resistant to NO compared with *B. pseudomallei* K96243 (28), and hence, the NosRPK pathway in *B. thailandensis* may be involved in other pathways rather than NO resistance.

Interestingly, although we found the *B. pseudomallei* Δ*nosP* and Δ*nosK* mutants had enhanced biofilm formation, in *B. thailandensis* E264, these mutants had reduced biofilm formation. The biofilm composition of these two species has been shown to significantly differ; *B. pseudomallei* biofilms have high levels of polysaccharide and low levels of extracellular DNA, whereas *B. thailandensis* E264 biofilms have low levels of polysaccharide and higher levels of extracellular DNA (46). *B. pseudomallei*, unlike *B. thailandensis* strain E264, has a polysaccharide capsule. The biofilms produced by capsule-deficient mutants of *B. pseudomallei* resemble those of *B. thailandensis* E264 biofilms by having increased levels of extracellular DNA, hence establishing a link between encapsulation and biofilm composition (46). A possible explanation for the contrasting effects on biofilm formation between the species of the *nosP* and *nosK* deletion mutants may be related to these differences in encapsulation and biofilm composition between *B. pseudomallei* and *B. thailandensis* E264.

### Implications of the Δ*nosK* or Δ*nosP* mutant phenotypes for infection

NosK and NosP regulate many infection-related phenotypes, including NO-resistance, biofilm formation, growth in nutrient-limited environments, and swimming motility. *B. pseudomallei* has adaptations to minimize NO exposure during infection; first, its LPS is weaker at inducing macrophage iNOS expression relative to the LPS of other Gram-negative pathogens (25), and second, it can suppress macrophage iNOS expression via a mechanism involving RpoS (26). The increased NO sensitivity of the Δ*nosK* or Δ*nosP* mutants could increase their susceptibility to immune clearance. Indeed, a *L. pneumophila* Δ*nosP* mutant displayed reduced virulence in macrophage models (20). Furthermore, *B. pseudomallei* likely experiences stress-inducing nutrient limitation and hypoxic conditions within infected tissues and may switch to respiratory denitrification to satisfy ATP demands, thus endogenously generating NO as a by-product (47), which could be more problematic for the Δ*nosK* or Δ*nosP* mutants due to their increased NO sensitivity. This increased NO sensitivity, coupled with the increased motility and biofilm phenotypes, makes it probable that the Δ*nosK* and Δ*nosP* strains will have altered virulence, and we intend to test this in future studies both *in vitro* and *in vivo*. As biofilm-dwelling *B. pseudomallei* evade immune defenses and show high levels of antibiotic resistance, NosK and NosP could be attractive targets for developing novel treatments that tackle biofilm formation and increase the susceptibility of *B. pseudomallei* to immune clearance or antimicrobial drugs that release NO, such as metronidazole (48).

## MATERIALS AND METHODS

### Culture conditions and growth parameters

Experiments using *B. pseudomallei* strains were performed in the University of Exeter’s biosafety level 3 approved laboratory. All *E. coli* and *B. pseudomallei* strains were grown at 37°C in LB medium, and where appropriate, shaken at 200 RPM unless otherwise stated. Antibiotics were used at the following concentrations: ampicillin (Amp) 100  µg/mL, kanamycin (Kan) 25  µg/mL, chloramphenicol (Cm) 50 µg/mL, and gentamicin (Gm) 50  µg/mL.

### Cloning

A full list of primers used in this study is outlined in Table S1. Genes were amplified from *B. pseudomallei* K96243 genomic DNA. For two-hybrid constructs, the T25 and T18 subunit of adenylate cyclase was added by cloning genes into either pKNT25 and pUT18, respectively, using either *Xba*I and *Eco*RI (*bpss1646*) or *Hind*III and *Bam*HI restriction sites (*bpss1647*). An N-terminal His-tag was added to *bpss1646* and *bpss1647* by restriction cloning into pQE60 using *Nco*I and *Bam*HI (*bpss1646*) or *Bam*HI and *Hind*III (*bpss1647*) restriction enzymes. *bpss1648* was cloned into pQE80-NusA to give a C-terminal His-tag using *Bam*HI and *Hind*III restriction sites. For making deletion constructs, ~500 bp upstream and downstream regions of *bpss1646* or *bpss1647* were PCR amplified and joined together using overlap extension PCR to make a ~1,000 bp fragment. This fragment was restriction cloned into pDM4 using *Xma*I and *Spe*I for both *bpss1646* and *bpss1647* deletion constructs. All constructs were transformed into calcium chloride competent *E. coli* strains by heat shock. For DNA propagation, pKNT25, pUT18, pQE60, and pQE80-NusA constructs were transformed into *E. coli* XL1 blue cells, whereas pDM4 constructs were transformed into *E. coli* GT115 cells.

### Bacterial adenylate cyclase two-hybrid assay

Calcium chloride competent DHM1 cells were transformed by heat shock with ~100 ng of pUT18 and pKNT25 constructs and selected for overnight at 37°C on an LB agar plate supplemented with Amp, Kan, and 2% glucose. As negative controls, each construct was also transformed alongside the empty inverse vector. A leucine zipper-positive control and a double empty vector-negative control transformation were also performed. For each transformation, a single colony was inoculated into a well of a 96-well plate containing LB media supplemented with Amp, Kan, and 2% glucose and then cultured for 4 h at 37°C, 200 RPM; 5 µL of the culture was spotted onto a nutrient broth agar (13 g/L nutrient broth [Thermo Fisher], 15 g/L agar) plate supplemented with 100 µg/mL X-gal, 0.1 mM IPTG, Amp, and Kan. Plates were incubated at 30°C for up to 72 h, and photographs were taken at regular time points.

### Protein expression and purification

Protein was purified essentially as previously described (49). Briefly, pQE60/80 constructs were transformed into M15(pRep4) cells. Single colonies were cultured overnight in LB media and then diluted 1:100 in 2YT media supplemented with Amp and Kan (and 20 µM hemin for pQE80-*bpss1647* transformants), and then grown at 37°C, 200 RPM. At OD_600_ of ~0.6, 100 µM IPTG was added, and the temperature decreased to 18°C. Cultures were grown at 200 RPM for 16 h before harvesting at 8,000 × *g* for 20 min at 4°C. Cell pellets were resuspended in 30 mL lysis buffer (49). Resuspended cells were lysed using a Sonics Vibra-Cell VCX 130 ultrasonic processor for 6 pulses of 20 s. Cell debris was pelleted by centrifuging the lysate at 19,000 × *g* for 30 min, and the resultant supernatant was filtered through a 0.4 µm filter. Filtered lysates were loaded onto a Ni-NTA agarose (Qiagen) column previously equilibrated with lysis buffer. Columns were washed with lysis buffer, and protein was eluted with elution buffer (lysis buffer containing 500 mM imidazole). Eluates of NosP had an orange tinge, indicating that the hemin had bound NosP.

### [γ-^32^P]ATP histidine kinase autophosphorylation and phosphotransfer assays

Autophosphorylation and phosphotransfer assays were performed essentially as previously described (49). Purified NosK and NosP were added to the TGMNKD buffer (49) and warmed to 37°C. The addition of 0.5 mM [γ-^32^P]ATP (3.7 GBq/mmol, PerkinElmer) commenced the autophosphorylation reactions; 10 µL samples of reaction mixture were taken at 1, 4, and 12 min, and the reactions were quenched by dispensing into 20 µL 1.5× SDS loading buffer (7.5% [wt/vol] SDS, 90 mM EDTA, 37.5 mM Tris-HCl, 37.5% [vol/vol] glycerol, 3% [vol/vol] β-mercaptoethanol, 0.05% [wt/vol] bromophenol blue, pH 6.8) and vortexing.

To assay for phosphotransfer, purified NosK was first incubated with 2 mM [γ-^32^P]ATP at 37°C in TGMNKD buffer for 45 min. NosR was then added to commence phosphotransfer reactions; 10 µL reaction samples were taken at 15, 60, and 240 s, and the reactions were quenched by vortexing with 20 µL 1.5× SDS loading buffer.

Samples from autophosphorylation and phosphotransfer assays were run on SDS-PAGE gels with a 5% acrylamide stacking gel and a 10% acrylamide resolving gel. Gels were rinsed with distilled H_2_O to remove background radiation and then exposed alongside standards to pre-erased phosphor sheets for 1 h. Exposed phospho sheets were imaged using a FUJIFILM FLA-7000 phosphorimager.

### Making unmarked deletions in *B. pseudomallei* K96243

Unmarked, in-frame *B. pseudomallei* K96243 deletion mutants were made using allelic exchange mutagenesis, essentially as previously described (50). Briefly, pDM4 deletion constructs were transformed into *E. coli* S17-1 λpir cells. Transformants were then used to conjugate the pDM4 construct into *B. pseudomallei* K96243. Merodiploids were selected for on LB agar containing Gm and Cm. Double recombinants were selected on 0 mM NaCl LB agar supplemented with 15% sucrose. Loss of Cm resistance was confirmed by replica plating suspected double recombinants onto LB agar supplemented with Cm and Gm and then onto LB agar supplemented with only Gm. Colonies which only grew on LB agar supplemented with Gm were genome prepped (Wizard Genomic DNA purification kit). Genomic DNA was used as a template for PCR confirmation of gene deletion. PCR products were sequenced to confirm in-frame deletion of the gene of interest.

### Growth assays

*B. pseudomallei* K96243 strains were grown overnight in 5 mL LB medium at 37°C, 200 RPM, and then standardized to OD_600_ 0.5 (~4 × 10^8^ CFU/mL) in either LB medium or M9SMM (1× M9 salts, 20 mM succinic acid, 2 mM MgSO_4_, 0.1 mM CaCl_2_). Standardized cultures were then diluted 1:10 in either LB medium or M9SMM in a 96-well plate to give a theoretical OD_600_ of 0.05. The plate was then incubated at 37°C in a Tecan Sunrise microplate reader with shaking (low intensity), and OD_600_ readings were taken at 15 min intervals.

### Congo red biofilm assays

Overnight *B. pseudomallei* cultures were diluted 1:100 in fresh LB medium, of which 5 µL was spotted onto the center of a Congo red agar plate (10 g/L tryptone, 5 g/L yeast extract, 10 g/L agar, with 40 µg/ml Congo red and 20 µg/mL Coomassie brilliant blue G, both solubilized in 70% EtOH). Plates were incubated at 37°C for 48 h, and then, the biofilms were allowed to mature at room temperature for 72 h before photographing.

### 96-well peg-lid biofilm assay

Biofilm formation was quantitatively assayed in a 96-well plate with a peg-lid (Innovotech) (51). Overnight cultures of *B. pseudomallei* strains were standardized to an OD_600_ 0.5 in LB media. Standardized cultures were then diluted 1:10 in fresh LB media and incubated statically at 30°C or 37°C for 24 h. The pegs were then washed by submerging into wells of a 96-well plate containing 200 µL PBS for 2 min, followed by drying at room temperature for 30 min. Biofilms were then stained by submerging the pegs for 20 min in a 96-well plate, with 200 µL per well of 0.1% crystal violet. The pegs were then washed again by three rounds of submerging into 96-well plates containing 200 µL PBS per well for 5 min. The peg lid was then dried at room temperature for 15 min. The crystal violet that had bound to the pegs was solubilized in a 96-well plate containing 200 µL per well of 95% ethanol for 20 min and quantified by measuring the absorbance at 550 nm in a Tecan Sunrise microplate reader. Biofilm formation from each independent experiment was normalized using the following equation:


$$\;\text{Normalized biofilm formation}=\frac{\text{Corrected }\;A550\;\text{nm}}{\text{Mean corrected wild-type }\;A550\;\text{nm}}$$

### Swimming motility assay

The swimming motility of *B. pseudomallei* strains was assessed using LB supplemented with 0.3% agar. The medium was poured into Petri dishes at least 4 h prior to inoculation. *B. pseudomallei* strains were grown overnight and standardized to OD_600_ 0.5. A pipette tip was dipped into the standardized culture and then stabbed into the center of the semi-solid agar plate. Plates were then incubated at 37°C for 48 h, and swim diameters were measured. Swim diameters from each independent experiment were normalized using the following equation:


$$\text{Normalized swim diameter}=\frac{\text{Swim diameter (cm)}}{\text{Mean wild-type swim diameter (cm)}}$$

### DETA NONOate challenge assays

Overnight cultures of *B. pseudomallei* strains were diluted in either cation-supplemented (22.5 µg/mL CaCl_2_, 11.25 µg/mL MgCl_2_) MHB or M9SMM to give a cell density of ~1 × 10^6^ CFU/mL. Next, 47.5 µL of MHB or M9SMM containing 2-fold serial dilutions of DETA NONOate at 2× the final desired concentration was prepared in a 96-well plate. The addition of 47.5 µL of ~1 × 10^6^ CFU/mL culture resulted in a final cell density of ~5 × 10^5^ CFU/mL (in line with Clinical and Laboratory Standards Institute recommendations) and the final DETA NONOate concentration being at 1×. Final DETA NONOate concentrations started at 500 µg/mL and decreased in a 2-fold stepwise manner until 3.9 µg/mL. A drug-free media column was included as an unchallenged control. Strains were also inoculated in either MHB or M9SMM containing 3.1 mM DETA (molar equivalent of the highest concentration of DETA NONOate used, 500 µg/mL) to confirm the growth inhibition observed is due to NO and not DETA. The 96-well plate was then incubated statically at 37°C for 20 h in a Tecan Sunrise microplate reader with OD_600_ measurements taken every 15 min. DETA NONOate-mediated growth inhibition data against each strain are presented as the percentage of growth relative to the growth of the untreated corresponding strain at 20 h using the equation:


$$\%\;\text{growth}=\left(\frac{\text{DETA NONOate treated strain OD}600\text{ at }t=n}{\text{Untreated strain OD}600\text{ at }t=20\text{ h}}\right)\times 100$$

The data were then fit with non-linear regression using GraphPad Prism, and IC_50_ values obtained.

## SUPPLEMENTAL MATERIAL

Supplemental material msphere.00423-25-s0001.pdf Fig. S1 to S4; Table S1.

Table S2 msphere.00423-25-s0002.xlsx Raw biofilm data for Fig. 5B.

ASM does not own the copyrights to Supplemental Material that may be linked to, or accessed through, an article. The authors have granted ASM a non-exclusive, world-wide license to publish the Supplemental Material files. Please contact the corresponding author directly for reuse.
