# Supplementary material for: A nitric oxide-sensing two-component system regulates a range of infection-related phenotypes in Burkholderia pseudomallei
Source: mSphere. 2025 Sep 17;10(10):e00423-25. doi: 10.1128/msphere.00423-25 (PMC12570481; doi:10.1128/msphere.00423-25)
Supplement: Supplemental material — Fig. S1 to S4; Table S1. [file msphere.00423-25-s0001.pdf]

Supplemental material

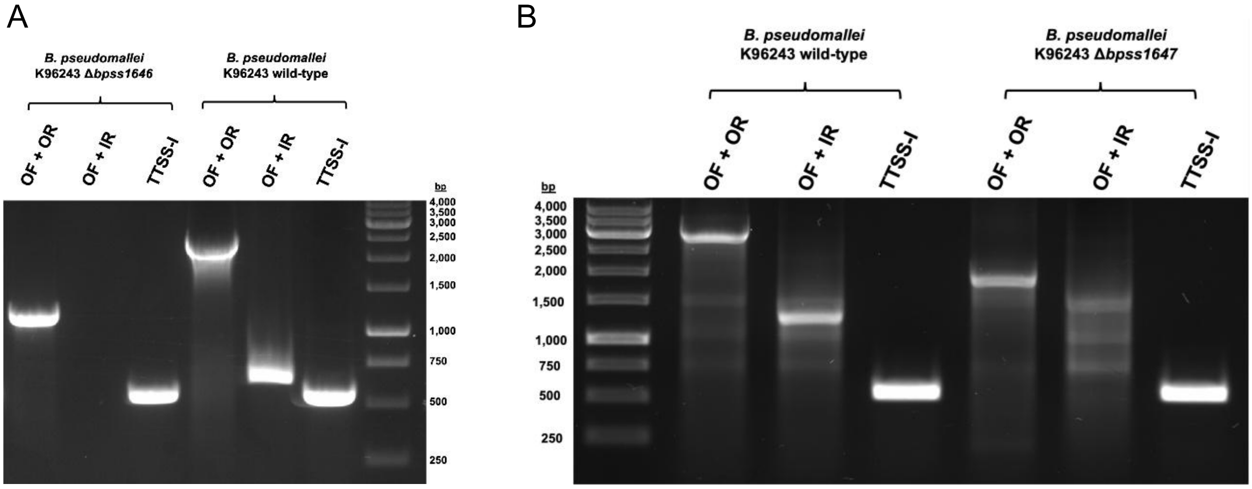

FIG S1 PCR confirmation of *bpss1646* (*nosK*) and *bpss1647* (*nosP*) deletion. OF and OR primer pairs were designed to amplify a DNA region that is outside of the flanking regions used in the deletion constructs. Owing to the deletion, the OF+OR PCRs will yield a shorter product for mutants than for wild-type revertants. The IR primers were designed against a region that was intended to be deleted in the mutants; PCRs involving IR were expected to fail for the deletion mutants. TTSS-I = BPTTF and BPTTR primers, which amplify *orf2* of the type 3 secretion system 1 (TTSS-1) of *B. pseudomallei*, act as a positive control and were not expected to differ between wild-type revertants and the mutants. (**A**) Deletion of *bpss1646*. OF - 1646KO_OF, OR - 1646KO_OR, IR - 1646KO_IR. Expected OF+OR band size for wild-type 2301 bp, mutant 1140 bp. (**B**) Deletion of *bpss1647.* OF – 1647KO_OF, OR - 1647KO_OR, IR - 1647KO_IR. Expected OF+OR band size for wild-type 3014 bp, mutant 1901 bp.

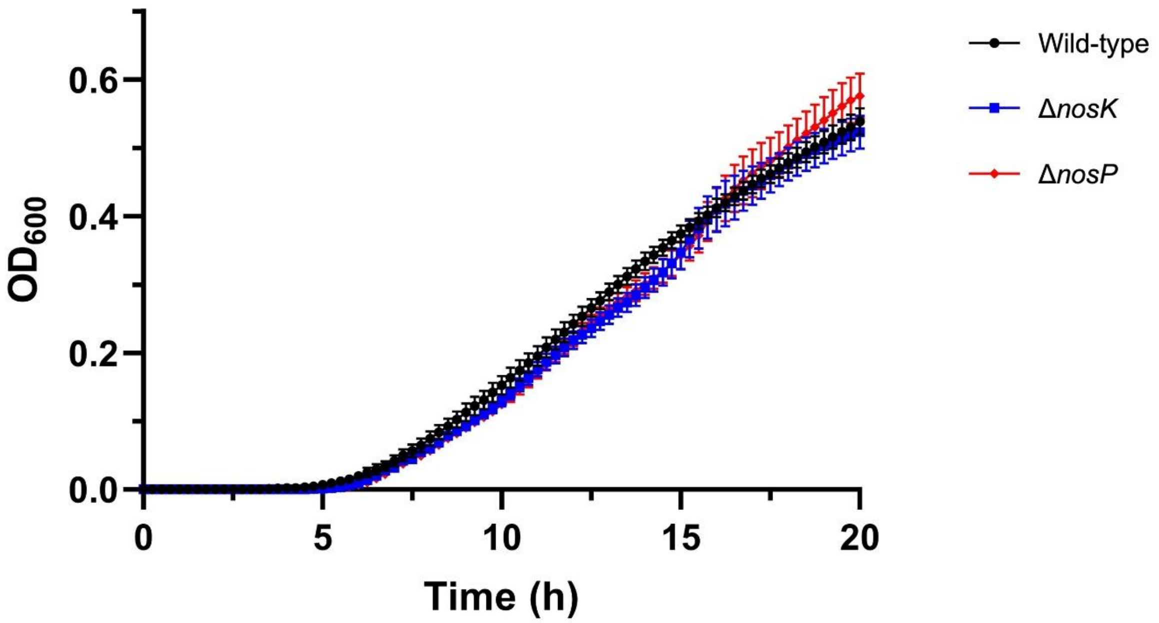

FIG S2 Growth curves of the *B. pseudomallei* K96243 wild-type, Δ*nosK* and Δ*nosP* strains in MHB. Data are the mean of 6 technical replicates from 3 independent experiments with error bars representing ± SEM.

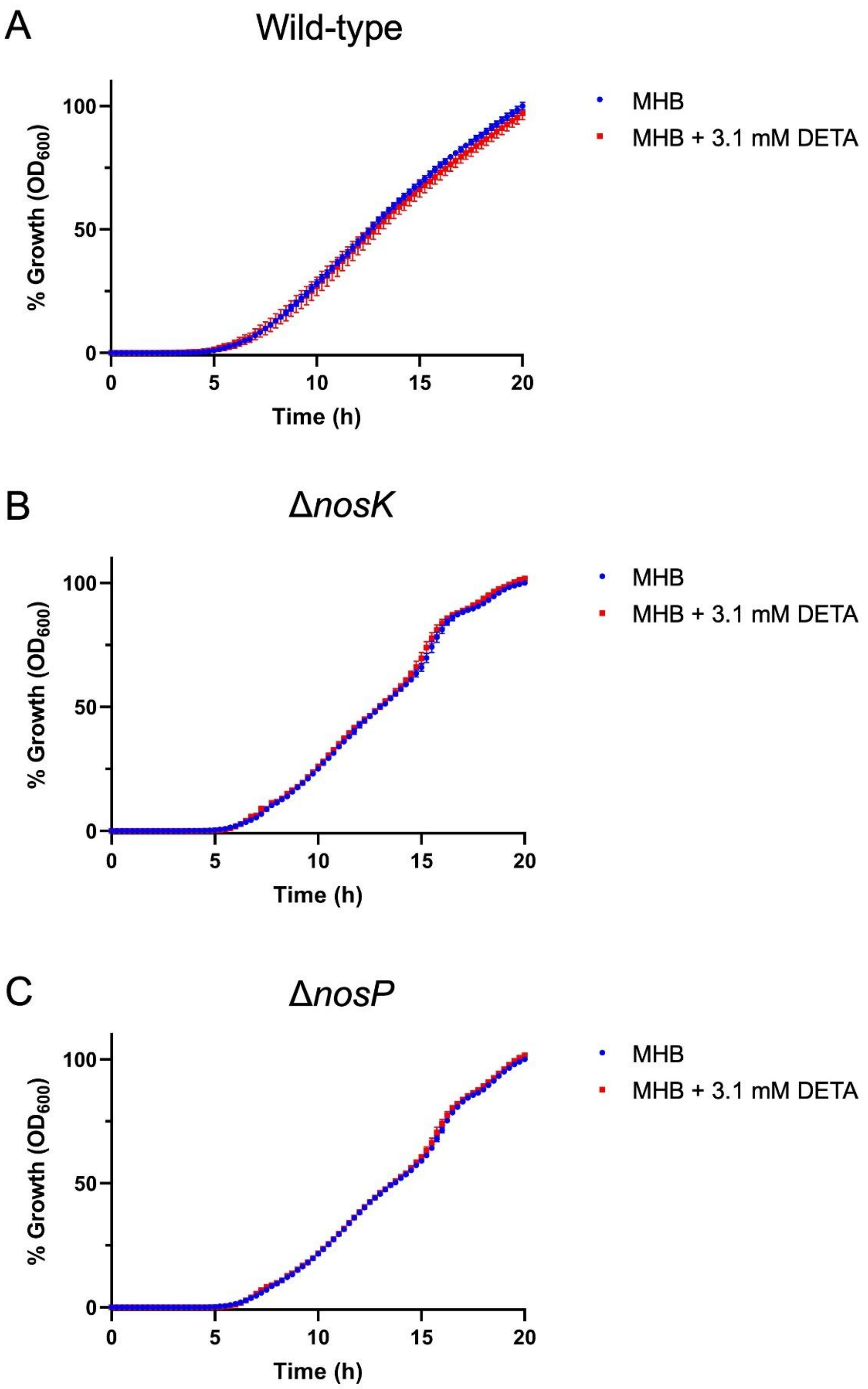

FIG S3 3.1mM DETA does not inhibit the growth of (**A**) the wild-type, (**B**) Δ*nosK* and (**C**) Δ*nosP* strains in MHB. Data are the mean of 4 technical replicates from 2 independent experiments and error bars represent ± SEM.

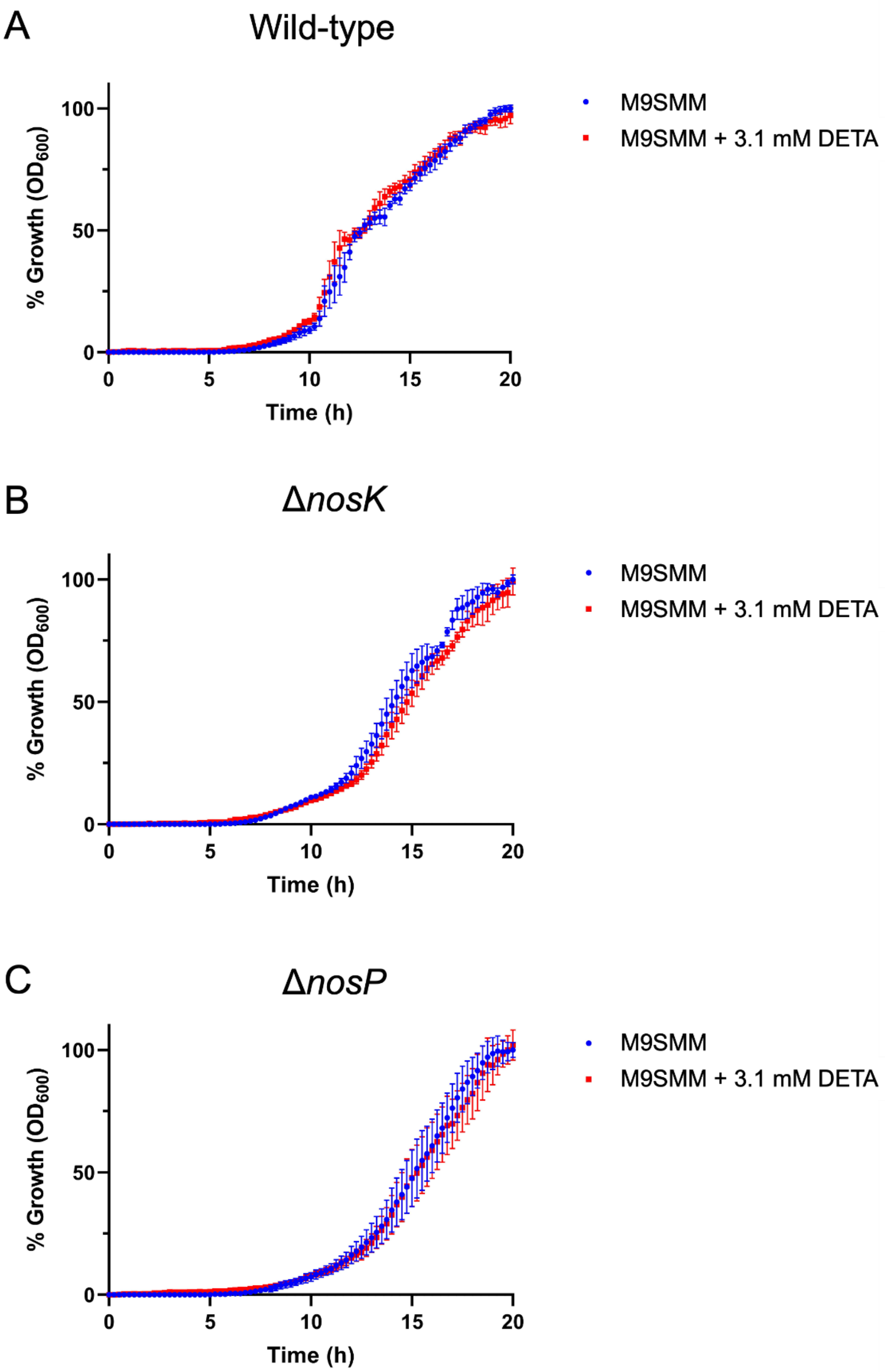

FIG S4 3.1 mM DETA does not inhibit the growth of (**A**) the wild-type, (**B**) Δ*nosK* and (**C**) Δ*nosP* strains in M9SMM. Data are the mean of 4 technical replicates from 2 independent experiments and error bars represent ± SEM.

TABLE S1 Table of primers used in this study

Primer name	Primer sequence
bpss1646F 2H	GCGTCTAGAGGTGGCTGAATCGAGTC
bpss1646R 2H	TCGAATTCCGGCCGGCCGCCGCATCCTGCAA
bpss1647F 2H	CCCCAAGCTTGATGAAGGGGAGATCTGTC
bpss1647R 2H	CGCGGATCCGGGCCACGGTCGGGCTCCGGC
bpss1646 pQE60 F	GATCCCATGGCTGAATCGAGTCTCGTCGAC
bpss1646 pQE60 R	CGCGGATCCGCCGGCCGCCGCATCCTGCAAC
bpss1647 pQE60 F	CGCGGATCCATGAAGGGGAGATCTGTC
bpss1647 pQE60 R	CCCCAAGCTTTCAGCCACGGTCGGGCTC
bpss1648 pQE80 F	CGCGGATCCATGAGCAAACGAGCGAAAGCG
bpss1648 pQE80 R	CCCCAAGCTTTCAGTCCAACATGCCGCG
1646UF	AGATATCCCGGGCCGCACCGTCAGCGAGATCAAC
1646UR	GATTAGCCAGATCTGAGACTCGATTCAGCCACGG
1646DF	AGTCTCAGATCTGGCTAATCCGCGCGCCCGTTC
1646DR	ATGCAAACTAGTATCGAACGATGGCTGTCAGGC
1647UF	AGATATCCCGGGTTCCGCTCGGCTCGCGCATTC
1647UR	GTGAGCGTAGATCTCCCCTTCATCAGTCGG
1647DF	GAAGGGGAGATCTACGCTCACCGGCATCGTC
1647DR	ATGCAAACTAGTCGCAGGTTCGAATCGACGAAG
1646KO_OF	ACGTTCAAGACCCAGCATTTCC
1646KO_OR	GGACGCCCAATCGAATCGAAC
1646KO_IR	GAGCGCACGATCTTGTTC
1647KO_OF	CCTGATCGTGTCCGATATGCGGATGCCGCGG
1647KO_OR	CGCGCAGATAGTCGAGATCGACC
1647KO_IR	GGCGGGAATCGTGGAAGACAAC
BPTTF	GCGCTTCAATCTGCTCTTTCCG
BPTTR	CAGGACGGTTTCGGACGAATC
